# Paramedics feelings and beliefs about COVID-19

**DOI:** 10.1192/j.eurpsy.2021.816

**Published:** 2021-08-13

**Authors:** H. El Kefi, K. Kefi, I. Bouzouita, W. Kabtni, A. Baatout, W. Krir, C. Bencheikh Brahim, A. Oumaya

**Affiliations:** Psychiatric Unit, Military Hospital of Tunis, tunis, Tunisia

**Keywords:** coronavirus, emotion, Culture, paramedic

## Abstract

**Introduction:**

The year 2020 was marked by the COVID-19 pandemic. Health services were overwhelmed by the demands for care. Paramedics were both the main actors in the fight and the victims of this pandemic.

**Objectives:**

The objective of our work was to assess paramedics’ feelings and beliefs about COVID-19.

**Methods:**

Descriptive and cross-sectional study including paramedics (nurses, orderlies) from the military hospital of Tunis. Data collection was carried out by a clinical psychologist. we studied paramedics’ feelings and beliefs about COVID-19.

**Results:**

A total of 161 paramedics agreed to answer our questionnaire. The average age was 37.73 years. The average number of years worked was 14.95 years. There were 85 women (52.8%) and 76 men (47.2%). The feelings about COVID-19 were anxiety in 127 (78.9%) paramedics and indifference in 34 (21.1%). The factors that could influence the spread of the pandemic were divine influence (25.5%), the organized fight against viruses (70.2%), our genetics (9.9%), mutations of the virus (6.8%), the BCG vaccine (21.7%), the Tunisian climate (5.6%), our food (13%).

**Conclusions:**

The beliefs and feelings of paramedics regarding COVID-19 are many and varied. These factors must be taken into consideration because they influence the involvement of paramedics in the fight against the virus and their compliance with health and safety rules.

